# Dose Variations Using an X-Ray Cabinet to Establish *in vitro* Dose-Response Curves for Biological Dosimetry Assays

**DOI:** 10.3389/fpubh.2022.903509

**Published:** 2022-05-17

**Authors:** Martin Bucher, Tina Weiss, David Endesfelder, Francois Trompier, Yoann Ristic, Patrizia Kunert, Helmut Schlattl, Augusto Giussani, Ursula Oestreicher

**Affiliations:** ^1^Department of Effects and Risks of Ionizing and Non-Ionizing Radiation, Federal Office for Radiation Protection (BfS), Oberschleißheim, Germany; ^2^Department of Medical and Occupational Radiation Protection, Federal Office for Radiation Protection (BfS), Oberschleißheim, Germany; ^3^Department of External Dosimetry, Institut de Radioprotection et de Sûreté Nucléaire (IRSN), Fontenay-aux-Roses, France

**Keywords:** biological dosimetry (biodosimetry), dose-response curves, X-ray cabinet, dose variation, dicentric chromosome (DC), micronuclei (MN), thermoluminescence dosimeter (TLD), EPR alanine dosimetry

## Abstract

In biological dosimetry, dose-response curves are essential for reliable retrospective dose estimation of individual exposure in case of a radiation accident. Therefore, blood samples are irradiated *in vitro* and evaluated based on the applied assay. Accurate physical dosimetry of the irradiation performance is a critical part of the experimental procedure and is influenced by the experimental setup, especially when X-ray cabinets are used. The aim of this study was to investigate variations and pitfalls associated with the experimental setups used to establish calibration curves in biological dosimetry with X-ray cabinets. In this study, irradiation was performed with an X-ray source (195 kV, 10 mA, 0.5 mm Cu filter, dose rate 0.52 Gy/min, 1^st^ and 2^nd^ half-value layer = 1.01 and 1.76 mm Cu, respectively, average energy 86.9 keV). Blood collection tubes were irradiated with a dose of 1 Gy in vertical or horizontal orientation in the center of the beam area with or without usage of an additional fan heater. To evaluate the influence of the setups, physical dose measurements using thermoluminescence dosimeters, electron paramagnetic resonance dosimetry and ionization chamber as well as biological effects, quantified by dicentric chromosomes and micronuclei, were compared. This study revealed that the orientation of the sample tubes (vertical vs. horizontal) had a significant effect on the radiation dose with a variation of −41% up to +49% and contributed to a dose gradient of up to 870 mGy inside the vertical tubes due to the size of the sample tubes and the associated differences in the distance to the focal point of the tube. The number of dicentric chromosomes and micronuclei differed by ~30% between both orientations. An additional fan heater had no consistent impact. Therefore, dosimetric monitoring of experimental irradiation setups is mandatory prior to the establishment of calibration curves in biological dosimetry. Careful consideration of the experimental setup in collaboration with physicists is required to ensure traceability and reproducibility of irradiation conditions, to correlate the radiation dose and the number of aberrations correctly and to avoid systematical bias influencing the dose estimation in the frame of biological dosimetry.

## Introduction

Suspected overexposure to ionizing radiation can be detected and quantified in biological dosimetry using specific biomarkers in peripheral blood (1). Thereby, the classification of the exposure level is based on established dose-response curves, which are derived from the analysis of blood samples irradiated *in vitro* under known conditions (2).

However, biological dosimetry includes much more than the conventional investigation of a single individual for possible overexposure to ionizing radiation. This was highlighted in a recent review paper by Ainsbury et al. on future applications in the field of emergency dosimetry, molecular epidemiological studies, personalized dosimetry after medical exposures or in the emerging sector of space tourism (3). In this context, the conventional and established methods of biological dosimetry, such as the analysis and quantification of dicentric chromosomes, translocations or micronuclei, will continue to play a central role in the future (4, 5). Various further developments have emerged in automated scoring (6–8) and the cooperation in international networks (9). In addition, the range of methods could be extended to premature chromosome condensation (10), γH2AX analysis (11, 12), gene expression analysis (13–15) or proteome analysis (16, 17), and multiple-parameter analysis could be used (18, 19).

Despite all these developments, dose-response curves will remain the basic requirement for correct and reliable dose estimation in case of an accidental overexposure. Although there have been efforts to harmonize and standardize biological dosimetry techniques since many years (20–23), these dose-response curves vary between laboratories not only due to differences in radiation quality and instruments employed, but also due to differences in the preparation and evaluation of the samples by the respective laboratory. Therefore, it is recommended that each laboratory establishes its own calibration curves from different radiation qualities to estimate doses for individuals that were potentially exposed to ionizing radiation (1, 2). The *in vitro* irradiation of blood samples and cells with different radiation sources and qualities is therefore an essential activity for biological dosimetry laboratories.

Due to the technical development in the past decades, nowadays there are X-ray cabinets, which are designed as high- or full- protection devices and have many advantages in terms of radiation protection regarding the use, maintenance and disposal. In contrast to the hemispherical symmetry of radionuclide sources, particularly the anode heel effect leads to inhomogeneities in the radiation field generated by X-ray tubes that influence the delivered dose (24–27). The common use of additional filtering to produce X-ray spectra of a desired quality or to remove unwanted energy ranges from the spectrum leads to additional inhomogeneities (28). In addition, several radiobiological studies have shown that the experimental setup itself affects the irradiation (28–31), as X-rays have usually lower photon energies than sealed sources and are thus easier attenuated (32). The resulting dose variation could have a significant impact on the yield of aberrations induced and thus on the dose estimation in biological dosimetry. Misinterpretation of the results might be the consequence.

Therefore, the aim of this study was to investigate how different experimental setups for the establishment of dose-response curves may affect the radiation dose delivered in an X-ray cabinet. For this purpose, the radiation dose effectively delivered was investigated with physical measurement methods [ionizing chamber, thermoluminescence (TL) dosimetry and electron paramagnetic resonance (EPR) alanine dosimetry], and correlated with biological effects quantified by dicentric chromosomes and micronuclei.

## Materials and Methods

### Irradiation Setup

The X-ray cabinet type RS225 (Xstrahl Limited, United Kingdom) equipped with a Varian NDI-226 X-ray tube (maximum tube voltage 225 kV, maximum current 13.5 mA, tungsten anode at angle 30°, inherent filtration 0.8 mm beryllium, radiation coverage 40°) was used for the irradiations. The irradiations in the experiments performed were done with 195 kV, 10 mA, an additional 0.5 mm copper filtering (Cu) and an additional 2.09 mm flattening filter made of aluminum (to compensate for field inhomogeneities), at a dose rate of 0.52 Gy / min and a distance of 50 cm from the focal point of the tube (focus-surface distance, FSD). The half-value layer (HVL) was determined at 195 kV, 10 mA and using 0.5 mm Cu additional filter material and corresponds to 1.01 mm Cu for the first HVL and 1.76 mm Cu for the second HVL. The average energy of the photon spectrum was calculated by SpekCalc (33–35) to be between 85.4 (without) and 86.9 keV (with flattening filter). Following an internal protocol closely related to the TG-61 protocol for the in-air method (36), dose rate was measured at the beam center at a radiation dose of 1 Gy and initially without considering possible effects of the experimental setup using a 0.6 cm^3^ Farmer chamber, PMMA/Al, type TM30010-1 (PTW Freiburg GmbH, Germany) and a UNIDOS E dosimeter (PTW Freiburg GmbH, Germany).

In order to assess the influence of experimental setup components, irradiations were performed in three different scenarios.

**Figure 1:**
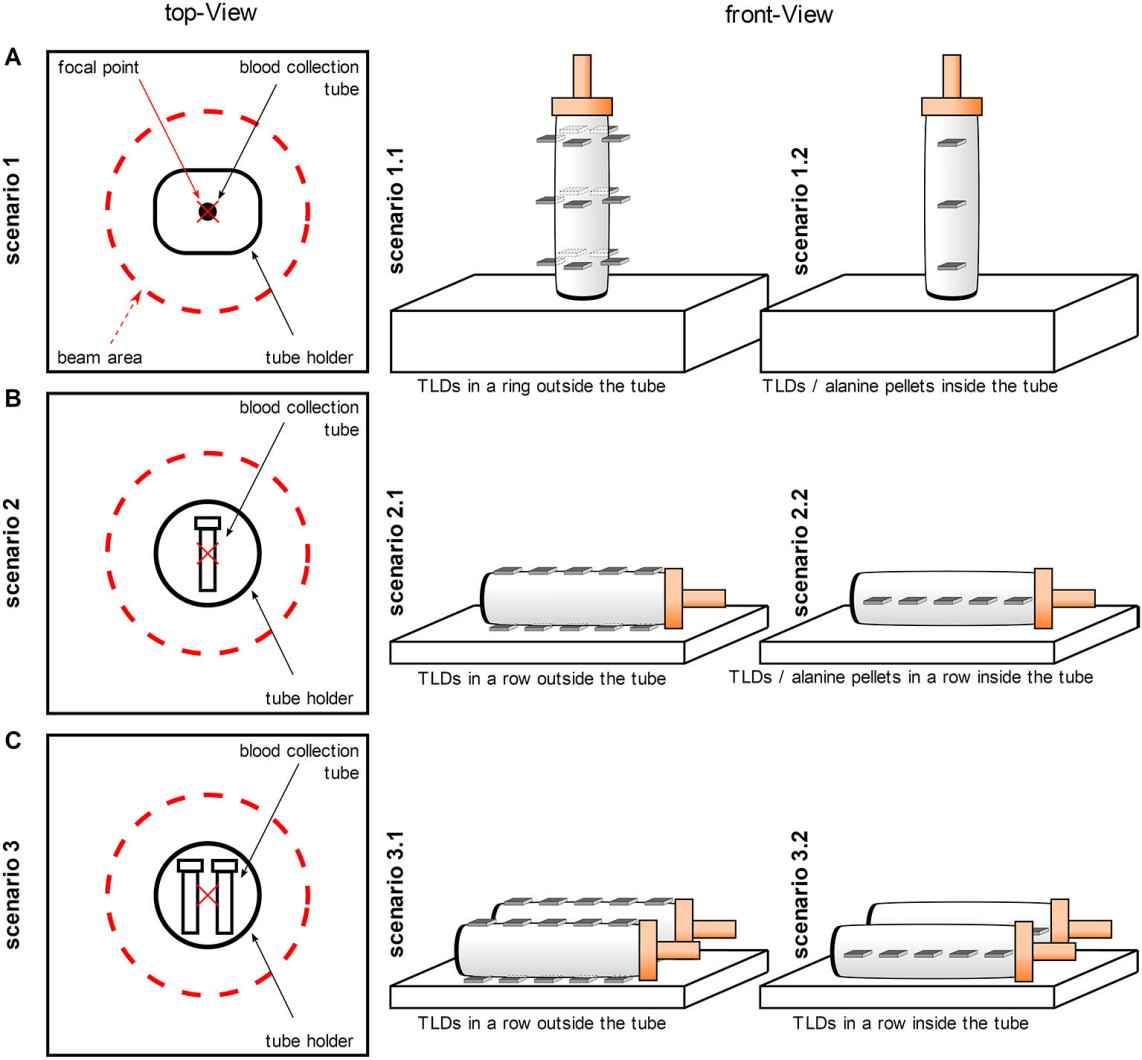
Schematic representation of the experimental setup. The irradiations were performed in three different scenarios. For all scenarios, the blood collection tubes were filled with water for physical dose measurements or with whole blood for biological dosimetry studies. **(A)** One blood collection tube was positioned vertically in the central position of the radiation field (scenario 1) and TLDs were placed in three rings on the outer surface of the tube (scenario 1.1) or TLDs or alanine pellets were placed in a row inside the tube (scenario 1.2). **(B)** One blood collection tube was placed horizontally in the center of the radiation field (scenario 2) and TLDs were placed in two rows above and below on the outer surface of the tube (scenario 2.1) or TLDs or alanine pellets were placed in one row inside the tube (scenario 2.2). **(C)** Two blood collection tubes were irradiated horizontally at the same distance from the central position of the radiation field (scenario 3) and the TLDs were placed in two rows above and below on the outer surface of the tube (scenario 3.1) or the TLDs were placed in one row inside the tube (scenario 3.2).

Scenario 1: One blood collection tube was placed in a 4.5 cm flat polystyrene tube holder and positioned vertically in the central position of the radiation field (Figure 1A). As a result of the shape of the tube holder, the tube was elevated so that the bottom of the tube was 2.2 cm closer to the source. Scenario 2 and 3: One blood collection tube (in scenario 3: two blood collection tubes) was placed horizontally in a 0.3 cm flat, circular tube holder (diameter 12.55 cm)**—**with a recess of 11 × 1 × 0.1 cm^3^ suitable for the blood collection tube**—**in the center of the radiation field. The tube holder was 3D-printed and made from Polylactic acid (PLA) to ensure reproducible and centered irradiation (Figure 1B). For scenario 3, both tubes had the same distance (0.85 cm) to the central position of the radiation field (Figure 1C).

To investigate the influence of a fan heater, a 27 × 19 × 7 cm^3^ fan heater (Xstrahl Limited, United Kingdom) was additionally positioned centrally 21 cm below the irradiation level in all scenarios.

The blood collection tubes (Sarstedt AG & Co. KG., Germany) had a volume of 10 mL and were 8.4 cm high and 1.7 cm in diameter. They were made from polypropylene with a high-density polyethylene (HDPE) cap, which was 2.5 cm high.

#### Irradiation of Thermoluminescence and EPR Alanine Dosimeters

For thermoluminescence measurements, 3.2 x 3.2 x 0.9 mm^3^ LiF:Mg,Ti chips (TLD100, Bicron-Harshaw, USA) were used. To compare the results of TL dosimetry, EPR measurements were performed with alanine pellets (Gamma Services Company, Germany). Irradiation was performed at 1 Gy for TL dosimetry or at 100 Gy for EPR alanine dosimetry at room temperature (RT) under the described conditions, and non-irradiated controls (blanks) were also processed to evaluate the background signal. The TL dosimeters (TLD) and alanine pellets were irradiated in water-filled blood collection tubes. For this reason, pellets were wrapped and sealed in a thin plastic foil to avoid partial damage and humidification of the pellet during the experiment, which causes an increase of the fading rate (37).

For scenario 1.1, five TLDs (representing five replicate measurements) were placed in three rings outside the tube, from the lid to the bottom of the tube (Figure 1A). The TLDs were positioned orthogonally to the incoming beam. The rings had a distance of 2.5 cm to each other so that the ring close to the lid and the ring close the bottom were a total of 5 cm apart and the ring close to the bottom was 2.3 cm apart from the bottom. The rings at different heights were irradiated one after the other and separately to avoid mutual shielding. For scenario 1.2, TLDs were also placed orthogonally to the incoming beam inside the tube at three different heights (distance 2.5 cm) from the lid to the bottom of the tube (Figure 1A). The irradiation of the TLDs in scenario 1.2 was repeated five times with different TLDs to obtain 5 replicates for each height. Similar to scenario 1.1, the TLDs at the different heights were irradiated separately to avoid mutual shielding. In another experiment, five alanine pellets were placed sequentially in a row inside the water-filled vertical tube and irradiated in one single run. For scenario 2, firstly, five TLDs were placed in two rows outside, below and above the tube (scenario 2.1). Secondly, five TLDs or five alanine pellets were placed in a row inside the water-filled horizontal tube (scenario 2.2; Figure 1B). For scenario 3, the TLDs were arranged in the same way as in scenario 2, with the difference that two blood collection tubes were irradiated simultaneously (scenario 3.1, 3.2; Figure 1C). In this scenario, no measurement was performed with alanine pellets.

In all scenarios, the blood collection tubes were filled with 8 mL water (at RT) and the heights of the liquids were 5 cm and 1.2 cm for vertical and horizontal tubes, respectively.

#### Dose Measurements (Calibration) With Ionization Chamber

In order to verify the results of TL and EPR dosimetry, measurements were performed using a 0.6 cm^3^ Farmer chamber, PMMA/Al, type TM30010-1 (PTW Freiburg GmbH, Germany) and an UNIDOS E dosimeter (PTW Freiburg GmbH, Germany). The ionization chamber was positioned at the different heights of the TLDs in the radiation field and on the different templates, but without blood collection tubes. At each position, five measurements were performed (corrected for temperature and air pressure).

The ionization chamber was initially calibrated by PTW Freiburg GmbH (Germany) under the following conditions and with the following results: beam quality ^60^Co gamma-rays (E_mean_=1.25MeV), 20°C, 1013.25 hPa, 50% relative humidity, absorbed dose to water, detector calibration coefficient 5.358 × 10^7^ Gy / C and electrometer calibration factor 1.000 ± 0.5%. In addition, a cross calibration of the ionization chamber and the electrometer against the X-Strahl standard equipment was carried out by the manufacturer X-Strahl Limited (United Kingdom). The X-Strahl standard equipment was calibrated to the National Physics Laboratory UK (NPL) secondary standard and has a calibration certificate provided by the NPL (certificate of calibration – 2010040216/5 – 22 March 2011). The cross-calibration was performed for X-rays at 195 kV, 10 mA and filtering with 0.5 mm Cu and relate to in-air calibrations. The determined calibration coefficient of the ionization chamber at 195 kV and 10 mA was 4.86 × 10^7^ Gy / C and was saved in the electrometer.

#### Irradiation of Blood Samples

Blood samples were *in vitro* irradiated in blood collection tubes with different orientations in the radiation field and additional setup components to evaluate the influence of the experimental setting (scenario 1–3; Figures 1A–C). Irradiation was performed with 1 Gy at room temperature (RT) or at 37 °C under the described conditions, and non-irradiated controls were included. All tubes were filled with 9 mL whole blood suspension and the suspension was 5 cm and 1.2 cm thick in vertical and horizontal tubes, respectively.

### Calibration and Readout of the Thermoluminescence Dosimeters and EPR Alanine Dosimeter

The TLDs were calibrated and read out as previously described (31).

Measurements of the alanine pellets were performed at RT with an X-band EPR spectrometer (Bruker E500, Bruker Corporation, USA) equipped with a high Q cavity. Recording of EPR spectra was performed with a microwave power of 2 mW, a modulation depth of 0.3 mT, a modulation frequency of 100 kHz and a magnetic field sweep of 12 mT. Ten EPR spectra were recorded for each pellet at minimum. Peak-to-peak amplitude of the central peak was reported for each spectrum and then related to dose. When repeating measurements of a pellet, between each measurement, the pellet was removed and replaced in the measurement tube in order to account for the contribution of the pellet positioning and spectrometry tuning in the uncertainty budget. To establish the calibration curves based on alanine spectra, pellets were irradiated at known doses (40, 60, 80, 100, and 130 Gy). Irradiations were performed with a linear accelerator at 10 MV (Clinac 2,100, Varian Medical Systems, USA) in a water tank (Surface Source Distance 90 cm and water depth 10 cm) in the reference conditions described in IAEA's code of practice (38) with dose rate in terms of absorbed dose in water of 2 Gy per minute. The beam was calibrated in terms of absorbed dose in water with an ionization chamber calibrated at the French primary lab (Laboratoire National Henry Becquerel, Saclay, France). A correction to consider the difference of water temperature during the different irradiations was applied. This effect was evaluated to be about 0.2% per °C (39). In order to correct the response of the dosimeters for the irradiations with ortho-voltage X-rays facilities, a set of pellets were irradiated in terms of dose in water at a dose of 5 Gy at the reference German facility (Physikalisch-Technische Bundesanstalt, Germany) with the following beams: TH-250, TH-200, TH-150, TH-120, and TH-70 (2). The characteristics of these X-ray beams are given in a report by Ankerhold (40). The energy correction factor applied was 1.10 ± 0.01. The total uncertainty on dose is estimated at 5.5% (*k* = 2).

### Blood Collection, Cell Cultivation, and Sample Processing

Peripheral blood samples from a healthy adult donor were obtained with signed informed consent, in heparinized tubes (Sarstedt AG & Co. KG., Germany) by venipuncture by physicians according to §15 of the code of medical ethics for physicians in Bavaria, Germany, following the principles of the Declaration of Helsinki.

To evaluate radiation-induced damage following irradiation, whole blood was incubated at 37°C for 2 h after irradiation to allow DNA damage repair. Subsequently, 0.5 mL of whole blood was transferred to culture tubes containing culture medium [RPMI-1,640 medium with Hepes, 10% FBS, 2.5% PHA, and 0.5% Pen/Strep (all from Pan-Biotech GmbH, Germany)] and samples were incubated at 37°C for a total of 48 h for the DIC assay and 72 h for the MN test. For the DIC assay, colcemid (Hoffmann-La Roche AG, Switzerland) was added to the culture at a final concentration of 0.08 μg/mL 24 h after setting up the culture. For the MN assay, cytochalasin B (Serva Electrophoresis GmbH, Germany) was added at a final concentration of 5.5 μg / mL 24 h after culture set up. Cell cultivation and sample preparation for the DIC assay and MN assay were performed following the IAEA recommendation and ISO standards (1, 21, 22).

After cultivation, cells were centrifuged (200 × g, 10 min) and treated hypotonic with 0.075 M potassium chloride (37°C, 15 min). The cells were again centrifuged (200 × g, 10 min) and fixed in methanol and acetic acid (3:1) (Honeywell, USA and Merck KGaA, Germany) for at least three times. The fixed cell suspension was stored at −18°C until slide preparation. For slide preparation, 20 μL cell suspension was dropped onto the slides and the quality was checked. For the DIC assay, cells were stained with 3% Giemsa's azur eosin methylene blue solution (Merck KGaA, Germany) / PBS for 5 min. The dried slides were mounted with Eukitt mounting medium (Sigma Aldrich GmbH, Germany). For the MN assay, slides were mounted with 16 μL Vectashield mounting medium containing 4′−6-diamidino-2-phenylindole (DAPI) (Vector Laboratories Inc, USA) for cell staining.

### Analysis of Dicentric Chromosomes and Micronuclei

Image acquisition and analysis was performed by microscopy using a Metafer Scanning System (MetaSystems Hard & Software GmbH, Germany) equipped with Zeiss AxioImager.Z2 epifluorescence microscope (objectives: Plan-Apochromat 10x/0.45 M27, 63x/1.4 Oil DIC M27, 100x/1.4 Oil DIC M27 and filters: DAPI) (Carl Zeiss AG, Germany), a CCD CoolCube Camera 1 m (MetaSystems Hard & Software GmbH, Germany), a transmitted or fluorescent light source XCite Exacte (Excelitas, USA), and Metafer4 Software (MetaSystems Hard & Software GmbH, Germany) (41).

The number of radiation-induced dicentric chromosomes was quantified semi-automatically and manually. A 90% area of prepared slides was scanned at 10x magnification, and metaphases were automatically identified by the metaphase finding module (MSearch) of the Metafer4 software (sensitivity 6.0). For manual scoring, only metaphase spreads with 46 centromeres were scored by eye at 63x or 100x magnification by a well-trained and experienced human scorer. Dicentric chromosomes were additionally validated by karyotyping using the Ikaros Karyotyping System combined with a deep learning algorithm for chromosome classification (MetaSystems Hard & Software GmbH, Germany). At least 1,000 cells were evaluated per sample. For semi-automatic scoring, the detected metaphases were automatically captured by the autocapture module (Autocapt) on the Metafer4 software at 63x magnification and the number of dicentric chromosomes was quantified using the DCScore module with an in-house developed analysis algorithm (classifier) (42, 43). Next, a human scorer evaluated the detected candidates and confirmed them as a dicentric chromosome or rejected them as a false positive. At least 4,000 cells were evaluated per sample.

The number of radiation-induced micronuclei was quantified semi-automatically and manually of at least 1,000 DAPI-stained binucleated cells, respectively. A 90% area of the prepared slides was scanned at 10x magnification and binucleated cells were automatically identified using an in-house developed classifier and Metafer4 software (sensitivity 5.0). In semi-automatic scoring, a human scorer additionally evaluated the detected micronuclei candidates and confirmed them as micronuclei or rejected them as false positives. In manual scoring, a human scorer analyzed each identified binucleated cell and quantified the number of micronuclei.

### Data Processing and Statistical Analysis

For TLD measurements, the median of all replicates for an experimental condition was calculated and compared. For the analysis of dicentric chromosomes and micronuclei, replicate slides for an experimental condition were shown separately in figures and the counts of replicate slides were combined for calculating mean values and statistical tests. Statistical tests to compare dicentric chromosome or micronuclei counts between settings were performed using generalized linear models (glm) with a quasi-Poisson distribution to allow for over-dispersed counts. R version 4.1.1 was used for all statistical analyses.

## Results

### Influence of the Experimental Setup on Radiation Doses

The influence of setup conditions and components to establish *in vitro* dose-response curves for biological dosimetry assays was analyzed by thermoluminescence, ionization chamber and EPR alanine dosimetry.

**Figure 2:**
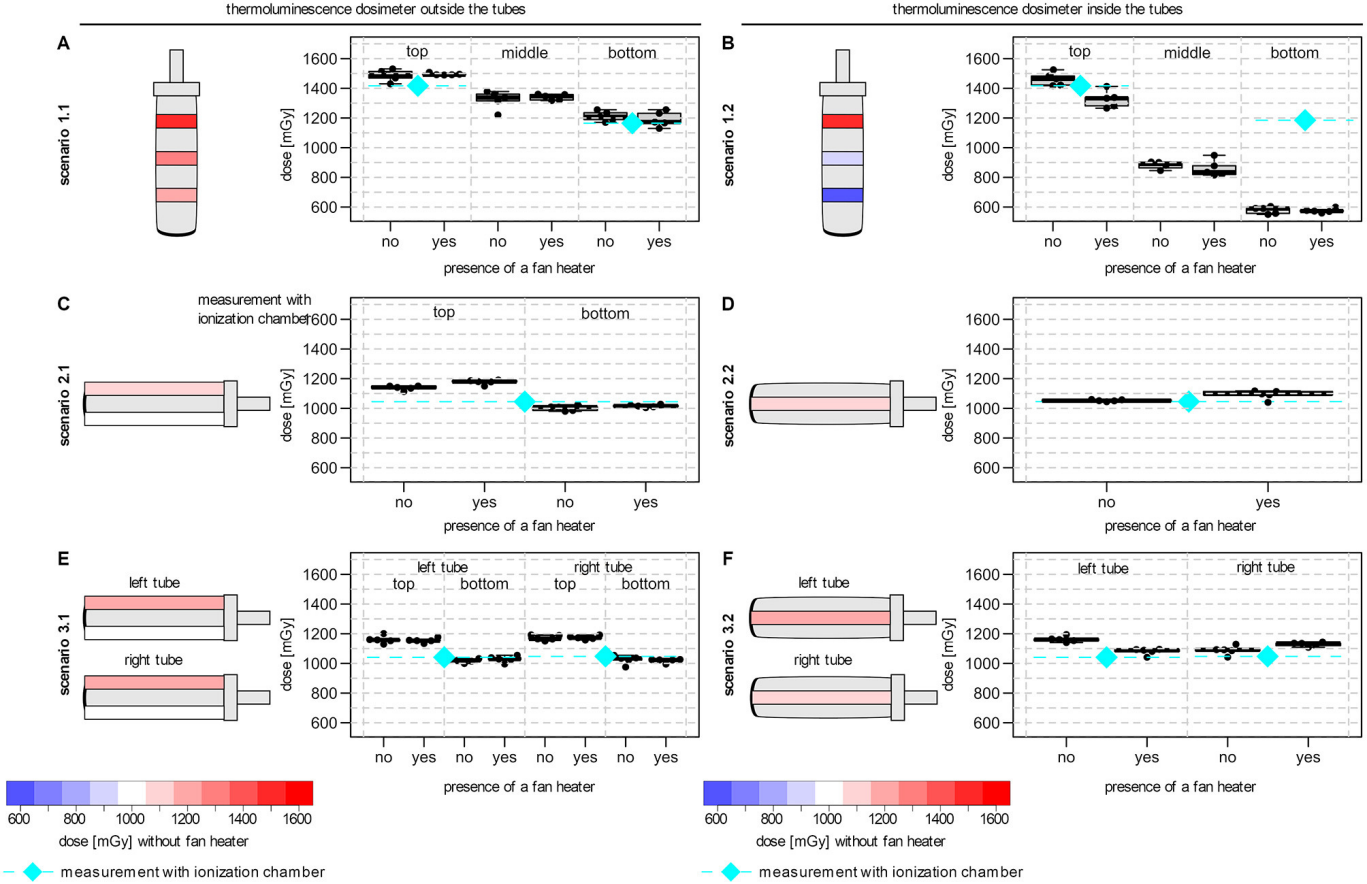
Effects of experimental setup associated with dose gradients on TLD dose measurement. The schematic orientation of the tube is shown with a color-coded representation of the dose variation (blue to red: low to high dose) and the median TLD doses for each position. **(A)** TLD measurements in three rings outside the vertical tube (scenario 1.1). **(B)** TLD measurements at three different heights inside the vertical tube (scenario 1.2). **(C)** TLD measurements above and below a single horizontal tube (scenario 2.1). **(D)** TLD measurements within a single horizontal tube (scenario 2.2). **(E)** TLD measurements above and below two horizontal tubes (scenario 3.1). **(F)** TLD measurements inside two horizontal tubes (scenario 3.2). All tubes were filled with water for the dose measurements. Irradiation was performed in the center of the beam area and in presence or absence of an additional fan heater. The median dose of the ionization chamber for the comparable positions (without fan heater) were also shown (diamond with dashed line in cyan). Boxplots showing the median, lower and upper quartiles of the number of the measured dose in mGy. Whiskers were derived as described in the default “boxplot” function of the statistic software R (version 4.1.1).

First, the radiation dose outside the water-filled blood collection tubes was measured. In these scenarios the planned radiation dose was 1 Gy. In scenario 1.1 (TLDs placed in three rings outside and from the lid to the bottom of the tube), where the blood collection tube was positioned in a vertical orientation, a substantial dose gradient was measured along the tube (Figure 2A). Within a total distance of 5 cm, the measured median TLD dose decreased from 1.49 Gy first to 1.34 Gy and further to 1.21 Gy. Thus, the dose at the upper and lower rings deviated considerably from the planned radiation dose of 1 Gy at FSD 50 cm. This decrease was confirmed by measurements using the ionization chamber (top 1.42 Gy vs. bottom 1.17 Gy, planned radiation dose 1 Gy). In contrast, when the tube was positioned horizontally in the center of the radiation field (scenario 2.1, Figure 2C), the dose gradient from TLDs above or below the tube was 1.14 vs. 1.01 Gy. When two tubes were irradiated simultaneously in horizontal orientation (scenario 3.1, Figure 2E), the observed dose gradient from above to below the tubes (left tube: 1.16 vs. 1.02 Gy; right tube: 1.17 vs. 1.03 Gy) was comparable to the single tube.

To determine the delivered dose inside the tube, TLDs were placed in the water-filled tubes. For the tubes in vertical orientation (scenario 1.2), the doses measured close to the lid were comparable between TLDs positioned inside (1.46 Gy; scenario 1.2) and outside (1.49 Gy; scenario 1.1) of the tube. However, in this scenario 1.2, a much stronger dose gradient was observed compared to scenario 1.1 (lid to bottom from 1.46 Gy to 0.90 Gy and 0.59 Gy; Figure 2B). In contrast, for the horizontal tubes, inside a median dose of 1.05 Gy was measured in scenario 2.2 (Figure 2D) and of 1.16 Gy and 1.09 Gy in scenario 3.2 for the left and right tube (Figure 2F), respectively. Measurements using the ionization chamber confirmed the median doses of scenario 2.2 (1.05 Gy) and scenario 3.2 (left tube: 1.05 Gy; right tube: 1.05 Gy).

The standard deviation for TLD measurements for all scenarios was always <5 % relative to the measured median TLD dose for all scenarios.

To compare the observed effects inside the tubes, additional irradiations of EPR alanine dosimeters were performed with water proof sealed alanine pellets. Here, a substantial dose gradient was also determined for the vertical tubes (lid to bottom from 134.68 Gy to 115.18 Gy to 99.9 Gy to 81.93 Gy to 70.02 Gy) and the dose at the upper and lower level deviated considerably from the planned radiation dose of 100 Gy at FSD 50 cm. In contrast, a median dose of 107 Gy was measured inside the horizontally tube.

The presence of an additional fan heater had no consistent and influencing effect on the delivered doses and the observed dose gradient in all scenarios (Figure 2).

### Influence of the Experimental Setup on Biological Effects

The influence of the experimental setup on the delivered dose was further investigated with regard to biological radiation effects. Whole blood was irradiated (1 Gy) in blood collection tubes in the specified three different scenarios (Figure 1) for which dicentric chromosomes and micronuclei were quantified.

**Figure 3:**
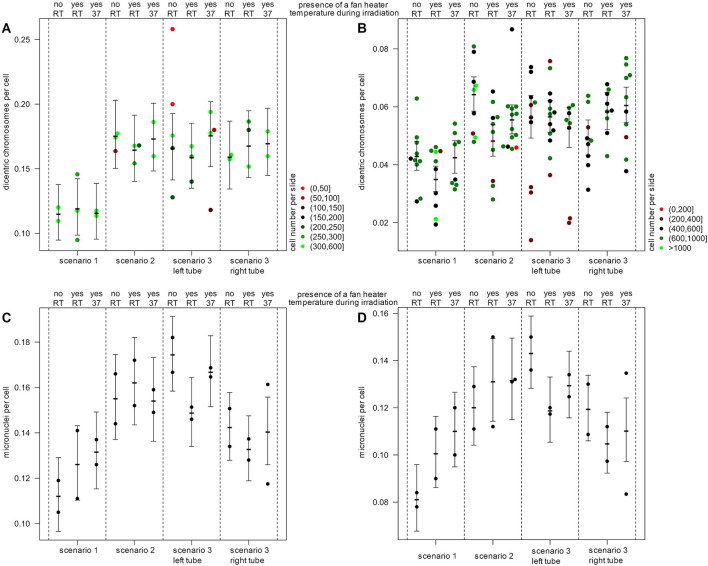
Effects of experimental setup on biological endpoints. The number of dicentric chromosomes for **(A)** manual and **(B)** semi-automatic quantification and the number of micronuclei for **(C)** manual and **(D)** semi-automatic quantification is illustrated. Shown is the mean number of damages per cell and per slide and for each scenario with the 95% confidence interval. The number of evaluated cells per slide is color-coded for dicentric chromosomes (red to green) for the analysis of micronuclei, 1,000 cells per slide were used. Blood samples were irradiated in the center of the beam area in the presence or absence of a fan heater and at different temperatures (37°C) or room temperature (RT).

For dicentric chromosomes, 13,074 cells on 36 slides and 74,654 cells on 119 slides were scored manually and semi-automatically, respectively. After irradiation, the formation of dicentric chromosomes was observed for all scenarios. Manual scoring revealed a significant lower number of dicentric chromosomes for the tube in vertical orientation (scenario 1: 0.11 dics/cell) in comparison to the horizontally positioned tubes (scenario 2: 0.18 dics/cell, *P* = 0.0007; scenario 3 left tube: 0.17 dics/cell, *P* = 0.0034 and right tube: 0.16 dics/cell, *P* = 0.013) (Figure 3A). No significant difference was observed between scenario 2 and both tubes of scenario 3 (left: *P* = 0.61; right: *P* = 0.41). As expected, for semi-automatic evaluation, in general a lower number of dicentric chromosomes per cell was detected (Figure 3B). Nevertheless, a lower number of dicentric chromosomes per cell was also observed for the vertical tube (scenario 1: 0.04 dics/cell) compared to the horizontal tubes (scenario 2: 0.06 dics/cell, *P* < 0.0001; scenario 3: left tube: 0.06 dics/cell, *P* = 0.003 and right tube: 0.05 dics/cell, *P* = 0.13). Here, a significant difference was observed between scenario 2 and the right tube of scenario 3 (left tube: *P* = 0.09; right tube: *P* = 0.0005).

In addition to the evaluation of dicentric chromosomes, the number of micronuclei was quantified in 29,879 cells on 24 slides each manually and semi-automatically. Micronuclei were observed following irradiation in all scenarios. By manual and semi-automatic evaluation, also fewer micronuclei were detected in the blood samples of the vertical tube (scenario 1: 0.11 MN/cell) compared to the horizontal tubes (scenario 2: 0.16 MN/cell, *P* = 0.0007; scenario 3 left tube: 0.17 MN/cell, *P* < 0.0001 and right tube: 0.14 MN/cell, *P* = 0.0071) (Figures 3C,D). Similarly, by semi-automatic evaluation fewer micronuclei were detected in the blood samples of the vertical tube (scenario 1: 0.08 MN/cell) compared to the horizontal tubes (scenario 2: 0.12 MN/cell, *P* = 0.0005; scenario 3 left tube: 0.14 MN/cell, *P* < 0.0001 and right tube: 0.12 MN/cell, *P* = 0.00015).

The presence of an additional fan heater and a temperature of 37°C during irradiation had no significant and consistent influence on the number of dicentric chromosomes or micronuclei.

## Discussion

Dose-response curves established *in vitro* are a fundamental requirement for a reliable dose estimation in biological dosimetry. For this purpose, X-ray cabinets are used more and more frequently, which offer many advantages in handling and radiation protection issues (28). The present study investigated the influence of the experimental setup of *in vitro* irradiated human blood samples on the radiation dose effectively delivered. All detected dose deviations are related to the deviations from the planned radiation dose, which were calculated from the dose rate in the isocenter. Therefore, the detected dose deviations are an indication of the effect of the experimental setup.

When blood collection tubes were irradiated in upright position (vertical orientation) a dose gradient of 280 mGy could be detected from the lid to the bottom outside of the tube by thermoluminescence dosimetry. The detected radiation dose was 21% to 49% higher than the planned dose at FSD 50 cm and the dose gradient outside the tube agrees with the inverse-square law for a point source. Measurements with an ionization chamber confirmed this observation. By contrast, a considerably higher dose gradient of 870 mGy was observed inside the tube by using TLDs, which cannot be predicted simply by the inverse-square law. The measured radiation dose deviated by −41% to +46% and measurements with alanine pellets confirmed a dose deviation of −30% to +35%. Since the dose measurements near the lid were comparable outside (1.49 Gy) and inside (1.46 Gy) the tube, an increasing shielding and absorption of photons by the liquid and the walls of tube leads to a decrease of the radiation dose, which might be even more pronounced for higher test tubes with denser material (30, 32). This deviation in the radiation dose consequently leads to a deviation in the dose rate for the different heights. In addition, it is important to mention that the mean values of dose measurements from TLD or alanine pellets used in the blood collection tubes are comparable to the planned radiation dose if the different heights are not considered and the dose gradient is not determined. This partial analysis may result in a misinterpretation and underestimation of the effects of the experimental setup.

On the contrary, when the blood collection tubes were irradiated in horizontal position, the dose gradient was lower and inside the tube the dose was only 50 mGy (+5%) higher compared to the desired planned dose at FSD 50 cm. This dose deviation was confirmed by EPR alanine dosimetry inside the tube (+7%) as well. Measurements by ionization chamber (without blood collection tubes) revealed a dose deviation of 5% at this position too. For the horizontal tubes, the dose gradient and dose deviations are consistent with the inverse-square law. Due to the horizontal orientation of the tube and a lower height of the liquid level (5 cm vs. 1.2 cm), less shielding and absorption effects occur. In this study, it was irrelevant in most cases whether one or two tubes were irradiated in the center of the radiation field, and is thus an indication of a homogeneous radiation field together with the results of an earlier study (31). At least for the horizontal tubes it is possible to correct the dose deviation, e.g., by adjusting the irradiation time or by specifying the true radiation dose, since in these cases the dose gradient has no significant influence. However, it must be kept in mind that this correction is limited to a specific scenario and time period and should always be determined by dosimetric tests in advance. The specification of general correction factors tempts to assume about an experimental setup without verifying them. In addition, the biological relevance of the deviation should be considered.

Due to technical variations and the biological variability of most biological assays, dose variations of 5–10% (depending on the sensitivity of the biological endpoints studied) may remain undetected and might therefore not be relevant for the analysis (44, 45). Thus, the maximum acceptable dose inaccuracy should be determined depending on the study method and the selected radiation dose, especially for quantitative studies, such as quantification of chromosomal aberrations and radiation-induced foci, gene expression or proteomic analyses (2, 44, 45). This observation of other studies is consistent with the results obtained here. As a consequence of the determined dose gradient, about 30% less dicentric chromosomes and micronuclei could be quantified in irradiated blood samples in vertical tubes by manual and semi-automatic analysis compared to horizontal tubes. However, the dose gradient not only affects the number of aberrant cells, but also influences their distribution. As a result of the approximately exponential dose decrease in the sample with increasing distance from the X-ray tube, as well as the shielding effects and the sinking of cells during the duration of irradiation, a large proportion of the cells are exposed to a lower dose than the rest of the cells. This results in an inhomogeneous irradiation of the blood samples and in a shifted distribution of aberrant cells. Typically, dicentric counts are Poisson distributed, and, in the field of biological dosimetry, a deviation from the Poisson distribution indicates an inhomogeneous or partial body exposure. For the dose gradient observed in this study, the probability for a significant deviation from the Poisson distribution will increase with increasing doses. Dose-response curves established in this way might lead to an overestimation of the exposure dose and a failure to detect partial body exposure.

In this study, all irradiations were performed in the center of the radiation field and only single sample vessels were irradiated. Previous studies have shown that many other factors, such as the field inhomogeneity, the energy spectrum of the X-rays, the dose rate and the shape, material, position and number of sample vessels or the volume of the cell suspension may have a significant effect on the absorbed dose (2, 28–31). In this study, the reduction in tube size and blood volume (e.g., 2– or 5 mL tubes) would contribute to a lower dose gradient and thus a more uniform radiation of the blood samples. Although it is often not necessary to irradiate a large number of samples simultaneously in biological dosimetry studies, the usage and positioning of different sample vessels and the presence of additional setup components, such as water baths or fan heaters, must be critically considered and require accurate dosimetric investigation prior to the start of the experiments. In addition to the substantial dose gradient through the experimental setup demonstrated in this study, it is highlighted that the dose rate at the isocenter cannot be used to determine the dose rate for a given experimental setup. The effects of the sample container, the medium, and possible field inhomogeneity have to be considered. Although dose rates were properly determined, the characteristics of X-ray cabinets may cause problems for short-term irradiations. Irradiations of <10 s should always be avoided, otherwise a difference between the targeted dose and the delivered dose may occur, which might range up to tens of percent (2).

The guidelines for the establishment of dose-response curves specified a temperature of 37°C during irradiation (1). By using an additional fan heater, no consistent effect on the radiation dose could be determined in the performed investigations, despite its size and the metallic surface that could lead to additional scatter radiation. Since the fan heater is placed about 21 cm below the beam area, it seems to be located sufficiently far away to not influence the radiation dose in this experimental setup.

A precise dosimetry of the experimental irradiation setup is always crucial for radiobiological studies to allow reproducibility of results and transfer of knowledge (28, 44). However, a lack of dosimetry in the establishment of dose-response curves may have consequences for the individuals involved in accident situations. Incorrect categorization of overexposed individuals in the case of mass-casualty incidence or discrepancies with additionally performed physical dose measurements may result in critical delay of urgent medical interventions of the affected individuals.

The following recommendations for the irradiation setup of blood samples are the result of the present and other studies (2, 28, 30, 31, 44, 46):

1. Dose-response curves for cytogenetic endpoints should always be established according to appropriate standards and guidelines (1, 21).
2. The settings of irradiation facilities should always be critically considered. Small changes in the irradiation modalities as well as in the experimental setup should always be critically evaluated, as they may result in significant effects. The sample vessels, the medium and the field inhomogeneity considerably influence the dose rate during irradiation.
3. Irradiation should always be performed in the center of the radiation field and sample vessels should be placed in horizontal orientation with a low liquid level to minimize shielding, absorption and inhomogeneities.
4. The establishment of dose-response curves should always be performed after robust preliminary investigations and accurate dosimetric studies, even when there is a time constraint or pressure to succeed.
5. Physicists should always be involved in study planning and design. Preliminary experiments are mandatory to avoid mistakes. If expertise is missing in the own institution or even in the country, then the knowledge and support of experienced biological dosimetry laboratories can be accessed, for example in the framework of networks (2).

## Data Availability Statement

The raw data supporting the conclusions of this article will be made available by the authors, without undue reservation.

## Ethics Statement

Ethical review and approval was not required for the study on human participants in accordance with the local legislation and institutional requirements. The patients/participants provided their written informed consent to participate in this study.

## Author Contributions

MB and UO designed the research. MB, YR, FT, and TW performed the research. AG, PK, UO, YR, HS, FT, and TW contributed to material and expertise. MB and DE analyzed the data, made figures, and wrote the manuscript. All authors discussed results, reviewed and revised the manuscript. All authors contributed to the article and approved the submitted version.

## Conflict of Interest

The authors declare that the research was conducted in the absence of any commercial or financial relationships that could be construed as a potential conflict of interest.

## Publisher's Note

All claims expressed in this article are solely those of the authors and do not necessarily represent those of their affiliated organizations, or those of the publisher, the editors and the reviewers. Any product that may be evaluated in this article, or claim that may be made by its manufacturer, is not guaranteed or endorsed by the publisher.
